# Genetic variation in the Y chromosome and sex-biased DNA methylation in somatic cells in the mouse

**DOI:** 10.1007/s00335-022-09970-z

**Published:** 2022-12-01

**Authors:** Enkhjin Batdorj, Najla AlOgayil, Qinwei Kim-wee Zhuang, Jose Hector Galvez, Klara Bauermeister, Kei Nagata, Tohru Kimura, Monika A. Ward, Teruko Taketo, Guillaume Bourque, Anna K. Naumova

**Affiliations:** 1 Department of Human Genetics, McGill University, Montréal, QC H3A 1C7 Canada; 2 Canadian Centre for Computational Genomics, Montréal, QC H3A 0G1 Canada; 3 Laboratory of Stem Cell Biology, Department of Biosciences, Kitasato University School of Science, 1-15-1 Kitasato, Minami-Ku, Sagamihara, Kanagawa 252-0373 Japan; 4 Institute for Biogenesis Research, John A. Burns School of Medicine, University of Hawaii, 1960 East-West Road, HonoluluHonolulu, HIHI 96822 USA; 5 The Research Institute of the McGill University Health Centre, Montréal, QC H4A 3J1 Canada; 6 Department of Surgery, McGill University, Montréal, QC H4A 3J1 Canada; 7 Department of Obstetrics and Gynecology, McGill University, Montréal, QC H4A 3J1 Canada

## Abstract

Several lines of evidence suggest that the presence of the Y chromosome influences DNA methylation of autosomal loci. To better understand the impact of the Y chromosome on autosomal DNA methylation patterns and its contribution to sex bias in methylation, we identified Y chromosome dependent differentially methylated regions (yDMRs) using whole-genome bisulfite sequencing methylation data from livers of mice with different combinations of sex-chromosome complement and gonadal sex. Nearly 90% of the autosomal yDMRs mapped to transposable elements (TEs) and most of them had lower methylation in XY compared to XX or XO mice. Follow-up analyses of four reporter autosomal yDMRs showed that Y-dependent methylation levels were consistent across most somatic tissues but varied in strains with different origins of the Y chromosome, suggesting that genetic variation in the Y chromosome influenced methylation levels of autosomal regions. Mice lacking the q-arm of the Y chromosome (B6.NPYq-2) as well as mice with a loss-of-function mutation in *Kdm5d* showed no differences in methylation levels compared to wild type mice. In conclusion, the Y-linked modifier of TE methylation is likely to reside on the short arm of Y chromosome and further studies are required to identify this gene.

**Supplementary Information:**

The online version contains supplementary material available at 10.1007/s00335-022-09970-z.

## Introduction

Mammalian males and females differ in many aspects of their phenotypes from anatomy to behavior. At the molecular level, sexual dimorphism is largely defined by sex differences in gene regulatory networks, and the sex-chromosome complement and gonadal sex are the major factors that shape sex differences in gene regulation (Burgoyne and Arnold 2016; Xu et al. 2002). To date, multiple studies have provided in-depth analyses and demonstrated the wide-ranging impacts of gonadal sex, gonadal sex-steroid hormones, and the pituitary growth hormone on gene expression and epigenetic modifications in the mouse liver (Conforto and Waxman 2012; Lau-Corona et al. 2017; Reizel et al. 2015). In contrast, the sex-chromosome complement appears to be critical for sex-biased gene expression in mouse preimplantation embryos, cultured cells, and the immune system with the X chromosome playing a major role (AlSiraj et al. 2019; Deegan et al. 2019; Engel 2018; Werner et al. 2017; Wijchers et al. 2010). However, the specific contribution of the Y chromosome to sex bias in gene regulation is still not well-understood (Deschepper 2020).

The mammalian Y chromosome is pivotal for sex determination, gonadal differentiation, and spermatogenesis. Several lines of evidence suggest that the Y chromosome also has a role beyond reproductive tissues [(Bellott et al. 2014), reviewed in (Hughes and Page 2015)] and influences regulation of autosomal genes as well as autosomal DNA methylation in somatic cells of non-reproductive organs (Deschepper 2020; Gatev et al. 2021; Ho et al. 2018; Wijchers et al. 2010; Zhuang et al. 2020). Indeed, several Y-linked protein-coding genes with X-linked paralogs that escape X-inactivation have been implicated in gene regulation and are expressed in somatic cells of non-reproductive organs in mice and humans. Y-linked lysine (K)-specific demethylase 5D (*KDM5D*/*Kdm5d*) is of particular interest as it encodes a lysine demethylase that demethylates histone H3K4me2/3 at gene promoter regions (Iwase et al. 2007). KDM5D-dependent histone H3K4 demethylation is associated with sex-biased gene expression in mice and sex-biased DNA methylation in humans (Grafodatskaya et al. 2013; Mizukami et al. 2019). Mutations in *Kdm5d* and its X-linked paralog *Kdm5c* affect heart development in mice (Kosugi et al. 2020). Another gene of interest is the ubiquitously transcribed tetratricopeptide repeat containing, Y-linked (*UTY/Uty*). UTY is critical during mouse preimplantation embryo development (Shi et al. 2021; Shpargel et al. 2012).

DEAD box helicase 3, Y-linked (DDX3Y) belongs to the family of ATP-dependent RNA-helicases (Sekiguchi et al. 2004) that are involved in the silencing of foreign DNA sequences, such as transgenes or endogenous retroviruses, in *Drosophila* (ElMaghraby et al. 2019; Lo et al. 2016) and may have conserved this function in other species. Eukaryotic translation initiation factor 2, subunit 3, structural gene Y-linked (*Eif2s3y*) is indispensable for spermatogenesis (Matsubara et al. 2015; Mazeyrat et al. 2001; Yamauchi et al. 2014) but also expressed in somatic cells (Werner et al. 2017). Moreover, four mouse Y-linked genes, zinc finger protein 2, Y-linked (*Zfy2*), *Uty*, *Ddx3y*, ubiquitin-activating enzyme, Chr Y (*Uba1y*) are expressed in embryonic stem cells and cannot be excluded as potential modifiers of DNA methylation or gene regulation at the very early stages of embryonic development (Werner et al. 2017).

To date, several studies have examined the influence of sex, sex-chromosome complement, gonadal sex hormone- and pituitary hormone-signaling pathways on DNA methylation in the mouse (Hao and Waxman 2021; McCormick et al. 2017; Reizel et al. 2015; Zhuang et al. 2020). However, most of these works did not analyze the impacts of X-dosage and Y chromosome separately. The objective of the present study was to evaluate the effects of the Y chromosome on autosomal DNA methylation. Our recent works have demonstrated that the presence of the Y chromosome may influence DNA methylation levels at autosomal loci in human fibroblasts and mouse livers (Ho et al. 2018; Zhuang et al. 2020). To explore the contribution of the Y chromosome to sex-biased DNA methylation, we analyzed DNA methylation in mice with different combinations of phenotypic sex and sex-chromosome complement using whole-genome bisulfite sequencing (WGBS) methylation analysis and identified Y-dependent differentially methylated regions (yDMRs) on autosomes. We find that most mouse yDMRs are located within transposable elements (TEs) and genetic variation in the Y chromosome modifies their DNA methylation levels.

## Materials and methods

### Mice and crosses

C57BL/6J mice were purchased from the Jackson Laboratory (Bar Harbor, Maine, USA).

B6.Y^TIR^ consomic mice were maintained in our colony (TT) by breeding of B6.Y^TIR^ males to C57BL/6J females. Sex-reversed females with two ovaries (XY.FT), true hermaphrodites with unilateral testis and contralateral ovary (XY.HT), and males with two testes (XY.MT), as well as XX females (XX.FT) were produced by crosses between C57BL/6J females and consomic males. “T” denotes mice originating from the crosses with B6.Y^TIR^ males. Liver, lung, heart, spleen, brain, tail, and testes were collected from adult mice at eight and 16 weeks of age and used for DNA or RNA extraction. Sex phenotypes were determined based on genital and gonadal phenotypes and were concordant with secondary sex characteristics. Gonadal sex of adult mice was confirmed at the time of organ collection.

B6.C3H/HeSn-Paf mice (referred to as *Paf* from this point on) were generated by backcrossing C3H/HeSn-Paf/J carriers of the patchy fur (*Paf*) mutation purchased from the Jackson Laboratory to C57BL/6J mice for several generations. In each generation, males that carried the *Paf* mutation were identified based on their hair loss phenotype and crossed to C57BL/6J females. Female offspring from these crosses were genotyped using RT-PCR for the *Xist* gene, which is expressed in XX females but not in XO females as described in (Alton et al. 2008). Liver samples from 8-week old XO females (XO.F) and their XX^*Paf*^ female (XX^*Paf*^.F) littermates from N6 and N7 generations were collected and used for DNA extraction.

*Kdm5d* knock-out mice carry a 2-nucleotide deletion in exon 1, which results in a frameshift mutation and premature termination of protein translation (Kosugi et al. 2020).

B6.NPYq-2 (XY*^X^*Sxr*^a^) males have a single X chromosome and Tp(Y)1Ct^*Sxr−a*^ (Cattanach et al. 1971) attached distal to the Y*^X^ PAR. These mice were first produced by intracytoplasmic sperm injection (ICSI) with sperm from X*Sxr*^a^Y*^X^ males on MF1 [National Institute for Medical Research (NIMR) colony] genetic background (Yamauchi et al. 2009), and then backcrossed to C57BL/6J for more than ten generations and maintained by ICSI. The sex reversal factor *Sx*r^a^ originates from an RIII strain Y chromosome.

DNA from different laboratory mouse strains was used for genotyping and pyrosequencing methylation analyses. F_1_ (C57BL/6J female x FVB/NJ male) mice (referred to as B6FVBF1) were generated in our mouse colony. The list of all mouse strains/crosses used in the study is provided in Table S1. The genotypes of the Y chromosome were inferred from the Mouse genomes project (MGP) data, when possible.

For most experiments, organ collection was performed on the same time of the day at Zeitgeber time (ZT) 7, i.e., seven hours after lights were switched on. All procedures were conducted in accordance with the guidelines set by the Canadian Council on Animal Care and were approved by the Animal Care Committee of the McGill University Health Center (Montreal, Quebec, Canada).

### DNA extraction from mouse organs and genotyping

DNA from mouse organs was extracted using a standard proteinase K phenol/chloroform procedure or by QIAamp Fast DNA Tissue Kit (Qiagen, NL).

Genetic sex was confirmed using PCR for Y-linked genes *Sry* (Table S2) or *Zfy* (as described in Amleh et al. 2000). Other genotypes were determined by PCR followed by Sanger sequencing to detect single nucleotide polymorphisms in selected Y chromosomal genes (Tables S1, S2). Sanger sequencing was done at the CES Genome Quebec (Montreal, QC, Canada).

### Detection of differentially methylated regions (DMRs) using WGBS data

Liver WGBS data from our previous study were used to identify differentially methylated regions (DMRs) (Zhuang et al. 2020). Differential methylation was detected using DSS (v2.32.0) (Park and Wu 2016) and methylKit (v1.10.0) (Akalin et al. 2012). CpG sites with low coverage (sequencing depth < 10) and that overlapped with common polymorphisms (SNP database 142) were excluded. Also, CpG sites with coverage > 500X were removed to account for PCR bias. DSS applied a 500-bp (default) smoothing window to estimate methylation levels of CpG sites and required a minimum length of 50 bp and containing at least three CpG sites to call a DMR. We also estimated DMRs with methylKit, with a tiling setting of 300 bp in length and 300 bp step-size windows. Differentially methylated tiles were detected with *q*-value < 0.05. For both DSS and methylKit, we only kept DMRs with methylation difference > 20%. We applied default parameters in both tools unless specified. We used the union of DMR results from both tools as our final list and DMRs from DSS were kept in the case of overlaps.

### Primer design and pyrosequencing methylation analysis

Primers for pyrosequencing methylation assays of DMRs were designed using the PyroMark Assay Design 2.0 Software (Qiagen, NL). For repetitive regions, we could not anchor primers to non-repetitive flanking sequences because the closest non-repetitive regions were farther than two kb from the differentially methylated CG sites. Hence, both PCR primers were located within the repeat and, in principle, could amplify more than one copy of the transposon. The list of primers for pyrosequencing methylation assays is provided in Table S3.

250 to 1000 ng of DNA per sample were treated with sodium bisulfite using EpiTect Bisulfite Kit (Qiagen, NL). Pyrosequencing was carried out using the PyroMark Q24 Advanced platform and PyroMark Q24 Advanced CpG Reagents (Qiagen, NL). Results were analyzed using the PyroMark Q24 Advanced software (Qiagen, NL).

### RNA extraction and gene expression analysis

RNA was extracted using TRIzol Reagent (Thermo Fisher Scientific, MA, US) according to the manufacturer’s instructions and followed by purification using the RNeasy MinElute Cleanup Kit (Qiagen, NL). CDNA was synthesized using one μg of RNA, oligo dT 12–18 primers and Moloney Murine Leukemia Virus reverse transcriptase (M-MuLV RT) (Thermo Fisher Scientific, MA, US). Quantitative RT-PCR (qPCR) was performed using Power SYBR Green PCR master mix (Thermo Fisher Scientific, MA, USA) and Eco Real-Time PCR System (Illumina, CA, USA). Gene expression levels were normalized to the housekeeping gene ribosomal protein L19 (*Rpl19*). Primers for expression analysis were designed using Primer3 software and checked with Bisearch (http://bisearch.enzim.hu) and IDT OligoAnalyzer (https://www.idtdna.com/pages/tools/oligoanalyzer). List of primers is provided in Table S2.

*Uty*, *Ddx3y*, *Eif2s3y*, and *Kdm5d* expression data were retrieved from our previously published RNA-seq results from the livers of C57BL/6N males and B6.Y^TIR^ mice (AlOgayil et al. 2021; Zhuang et al. 2020).

### Statistical analysis

Non-paired *t*-test, one- or two-way ANOVA tests followed by multiple comparisons (GraphPad Prizm 9.0.1 software) were used to determine the statistical significance of pyrosequencing methylation and RT-qPCR data.

## Results

### Y-dependent autosomal DNA methylation in the mouse

To identify DMRs that were associated with the presence of the Y chromosome (yDMRs) in the mouse liver, we analyzed WGBS data from females with different sex-chromosome complements, i.e., XX females (XX.FT, *n* = 3), sex-reversed XY females (XY.FT, *n* = 3), and females with monosomy X (XO.F, *n* = 3), as well as males (XY.MT, *n* = 3) (as described in Zhuang et al. 2020, data available from GSE217666). The WGBS data were filtered to exclude regions of low coverage and common polymorphisms (see Methods and Zhuang et al. 2020). After filtering, an average of about one third (15 million) of the 43822587 CGs sites of the reference mouse genome were available for analysis.

Autosomal DNA methylation levels from the WGBS data were contrasted in three comparisons: XY.FT vs XO.F, XX.FT vs XY.FT, and XX.FT vs XY.MT using two different analysis tools, DSS and methylKit (Zhuang et al. 2020). These tools detect regions that may contain from one to multiple differentially methylated cytosines (DMCs). The XX.FT vs XY.MT comparison identifies both sex-chromosome complement dependent as well as gonadal-sex dependent DMCs and DMRs. The XX.FT vs XY.FT comparison identifies DMCs and DMRs that are sensitive to the X chromosome dosage and/or the presence of the Y chromosome, whereas the XY.FT vs XO.F comparison detects Y chromosome dependent DMRs. The XO.F mice are not congenic and carry C3H alleles on the C57BL/6J genetic background. Hence, the XY.FT vs XO.F comparison would detect regions that are sensitive to variation in the genetic background too. To circumvent the impact of genetic variation and reduce the chance of false-positive results, Y-dependent DMRs (yDMRs) were identified as those that overlapped between three comparisons, the XY.FT vs XO.F, XX.FT vs XY.FT, and XX.FT vs XY.MT. In total, 785 autosomal DMRs were found in the XY.FT vs XO.F comparison, 802 DMRs—in the XX.FT vs XY.FT, and 2854—in the XX.FT vs XY.MT comparison. Nineteen autosomal DMRs (two with higher and 17 with lower methylation in XY mice) were common between the three comparisons and were defined as yDMRs. The mean length of autosomal yDMRs was 346.5 bp, with maximum length of 892 and minimum length of 137 bp. The average GC content of yDMRs was 42%. Seventeen of these (89%) overlapped with transposable elements (TEs), eight LINEs, one SINE, and eight LTRs and only two resided in non-repetitive regions (Table 1). All yDMR-associated LINE1 belong to the families of young and transcriptionally active L1_Md transposons, and three of these yDMRs (on chromosomes 12, 14, and 17) map to the promoter region of the open reading frame 1 (ORF1) of L1_Md.

**Table 1:** List of yDMRs in mouse liver

Chr	Start	End	Size of yDMR	Hyper/Hypo methylation compared to XX	Repeat over DMC	Family	Number of DMCs	Validated using pyrosequencing
2	103766401	103766700	299	Hypomethylated	No repeat element		1	Yes
2	155020006	155020478	472	Hypermethylated	LTR (ERVB4_1-I_MM-int)	ERVK	6	Yes
2	155023201	155023500	299	Hypomethylated	LTR (ERVB4_1-I_MM-int)	ERVK	2	Yes
2	155024401	155024700	299	Hypomethylated	LTR (ERVB4_1-I_MM-int)	ERVK	3	
2	155024701	155025000	299	Hypomethylated	LTR (ERVB4_1-I_MM-int)	ERVK	3	
3	57683399	57683826	427	Hypomethylated	LTR (MMETn-int)	ERVK	2	
3	129030879	129031129	250	Hypomethylated	LINE (L1Md_T)	L1	2	
3	129668867	129669272	405	Hypomethylated	LTR (RLTR45-int)	ERVK	3	
3	129672722	129673189	467	Hypomethylated	LTR (RLTR45-int)	ERVK	2	
4	61783240	61784131	891	Hypermethylated	SINE (MIR)	MIR	1	
4	82014619	82014755	136	Hypomethylated	LINE (L1Md_A)	L1	1	
5	151629536	151629952	416	Hypomethylated	LINE (L1Md_F2)	L1	1	
6	13714801	13715100	299	Hypomethylated	No repeat element		1	Yes
8	24147191	24147429	238	Hypomethylated	LINE (L1Md_F2)	L1	3	
11	31039801	31040100	299	Hypomethylated	LINE (L1Md_T)	L1	1	
12	81394600	81394904	304	Hypomethylated	LINE (L1Md_F2)	L1	4	
14	12252641	12252983	342	Hypomethylated	LINE (L1Md_A)	L1	4	
16	21107101	21107400	299	Hypomethylated	LTR (IAPEz-int)	ERVK	1	No
17	14014391	14014733	342	Hypomethylated	LINE (L1Md_A)	L1	3	

**Fig. 1:**
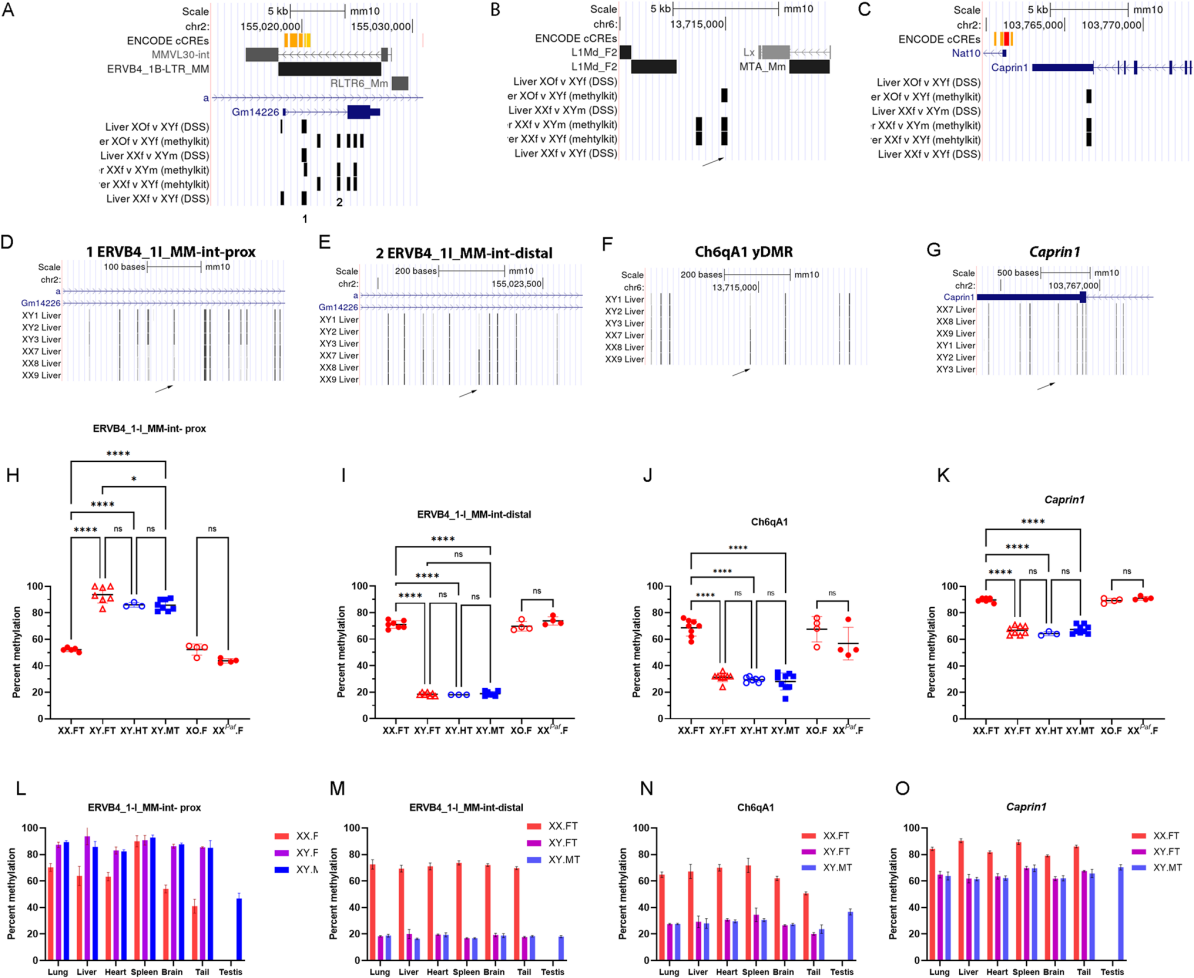
Validation and analysis of organ-specificity of yDMRs. **A** Genomic positions of yDMRs overlapping with intron 1 of the agouti gene. **B**, **C** Genomic positions of the Ch6qA1 (**B**) and *Caprin1* (**C**) yDMRs. Tracks show positions of DMRs from the XY.FT vs XO.F, XX.FT vs XY.FT, and XX.FT vs XY.MT comparisons based on WGBS data. **D–G** Positions of the DMCs that were interrogated by pyrosequencing assays. The XX7, XX8, XX9, XY1, XY2, and XY3 liver tracks show positions of CG sites, and the intensity of the tick reflects methylation level (WGBS data from Zhuang et al. 2020) in XX.FT and sex-reversed XY.FT female livers. **H–K** Validation of yDMRs in XX.FT (*n* = 7–8), XY.FT (*n* = 7–8), XY.HT (*n* = 3–7), XY.MT (*n* = 8–9), XX.^*Paf*^.F (*n *= 4), and XO.F (*n* = 4) livers. XY mice have significantly different levels of DNA methylation compared to XX or XO mice. Statistically significant differences are shown as asterisks ****p < 0.0001, *ns* not significant (one-way ANOVA followed by multiple testing with Tukey’s correction). **L-O** Methylation of yDMRs in organs of XX.FT, XY.FT, and XY.MT mice. Differences between XX and XY mice are statistically significant in all organs with the exception of ERVB4-prox yDMR in the spleen. **A**–**G** features shown in the context of the UCSC browser (mm10)

For five yDMRs, including two yDMRs located within the ERVB4_1I_MM-int (referred to as ERVB4-proximal and ERVB4-distal from this point on) located in intron 1 of the agouti gene on chromosome 2, the yDMR located within the intracisternal A particle IAPz-int repeat on chromosome 16, and two in the non-repetitive regions of chromosomes 2 and 6, we were able to design pyrosequencing primers (Tables 1, S3; Fig. 1A–G). Pyrosequencing methylation analysis in independently collected samples of XX.FT, XY.FT, XY.HT, XY.MT, XX^*Paf*^.F, and XO.F livers showed that methylation of the ERVB4, Ch6qA1, and *Caprin1* yDMRs was Y chromosome dependent, as methylation levels were different in mice that carried the Y chromosome, independent of their gonadal sex (Fig. 1H–K). The IAPz-int region on chromosome 16 showed no significant differences in methylation levels between XX.FT and XY.FT females and was not used in further experiments.

### Y-dependent DNA methylation is present across different mouse organs

To determine whether the effect of the Y chromosome on methylation was limited to liver, we tested yDMR methylation in other organs from the same mice: lung, heart, spleen, brain, tail, and testis. These organs represent the three germ layers of the mouse embryo with brain originating from the ectoderm, heart and spleen from the mesoderm, and liver and lung from the endoderm. The tail consists of skin (ectoderm) and bone (mesoderm), whereas adult testis consists of somatic (mesoderm) and germ cells. The ERVB4-proximal yDMR had higher methylation in most organs of XY compared to XX animals, whereas no differences between males and females were found in the spleen (Fig. 1L). In the testis and tail, methylation levels were lower than in other organs. The ERVB4-distal, Ch6qA1, and *Caprin1* yDMRs had lower methylation in all organs of XY compared to XX mice (Fig. 1M–O).

### Genetic variation in the Y chromosome influences yDMR methylation levels

Genetic variation may influence DNA methylation levels in *cis*, by affecting transcription factor binding sites, or *trans* through genetic variants in transcription factors or epigenetic modifiers that alter their ability to bind DNA or their binding specificity. To evaluate the influence of genetic variation on Y-dependent methylation, we first examined yDMR methylation levels in females and males from seven laboratory mouse strains, C57BL/6J, C57BL/6N, C3H (C3H/HeJ and C3H/HeH), DBA1/J, MOLF/EiJ, CAST/EiJ, FVB/NCrl, as well as F_1_(C57BL/6J female x FVB/NJ male) mice that carry the FVB/NJ Y chromosome. The subspecific origin of the Y chromosome in each of these strains was inferred from the Mouse genomes project (MGP) data and data from (Chang et al. 2017; Hughes and Page 2015; Morgan et al. 2015) (Table 2). To confirm or establish the genetic origin of the Y chromosome for strains not represented in the above sets of data, we genotyped male mice for four Y-chromosomal SNPs (Table S1).

**Table 2:** Characteristics of mouse strains used in the study

Strain	Origin of the strain according to whole exome sequencing data (Y chromosome not included) Chang et al. (2017)	Origin of the Y chromosome based on the phylogenetic tree from Morgan et al. (2015)	Origin of the Y chromosome based on SNPs from MGP and/or genotyping in our lab (Table S2)	Presence of ERVB4 insertion in the intron of the agouti gene based on data from Tanave et al. (2019)
B6.Y^TIR^	Nt	Nt	*domesticus*	Present
C3H/HeJ	*domesticus*	*musculus*	Nt	Absent
C3H/HeH	*domesticus*		*musculus*	Absent
C57BL/6J	*domesticus*	*musculus*	*musculus*	Present
C57BL/6N	*domesticus*	*musculus*	*musculus*	Present
CAST/EiJ	*castaneus*	*castaneus*	*castaneus*	Absent
DBA1/J	*domesticus*	*musculus*	*musculus*	Present
FVB/NCrl FVB/NJ	*domesticus*	*domesticus*	*domesticus*	Absent
MOLF/EiJ	*musculus and castaneus*	Nt	*domesticus*	Absent

*Nt* not tested, *domesticus* Mus musculus domesticus, *musculus* Mus musculus musculus*, castaneus* Mus castaneus

**Fig. 2:**
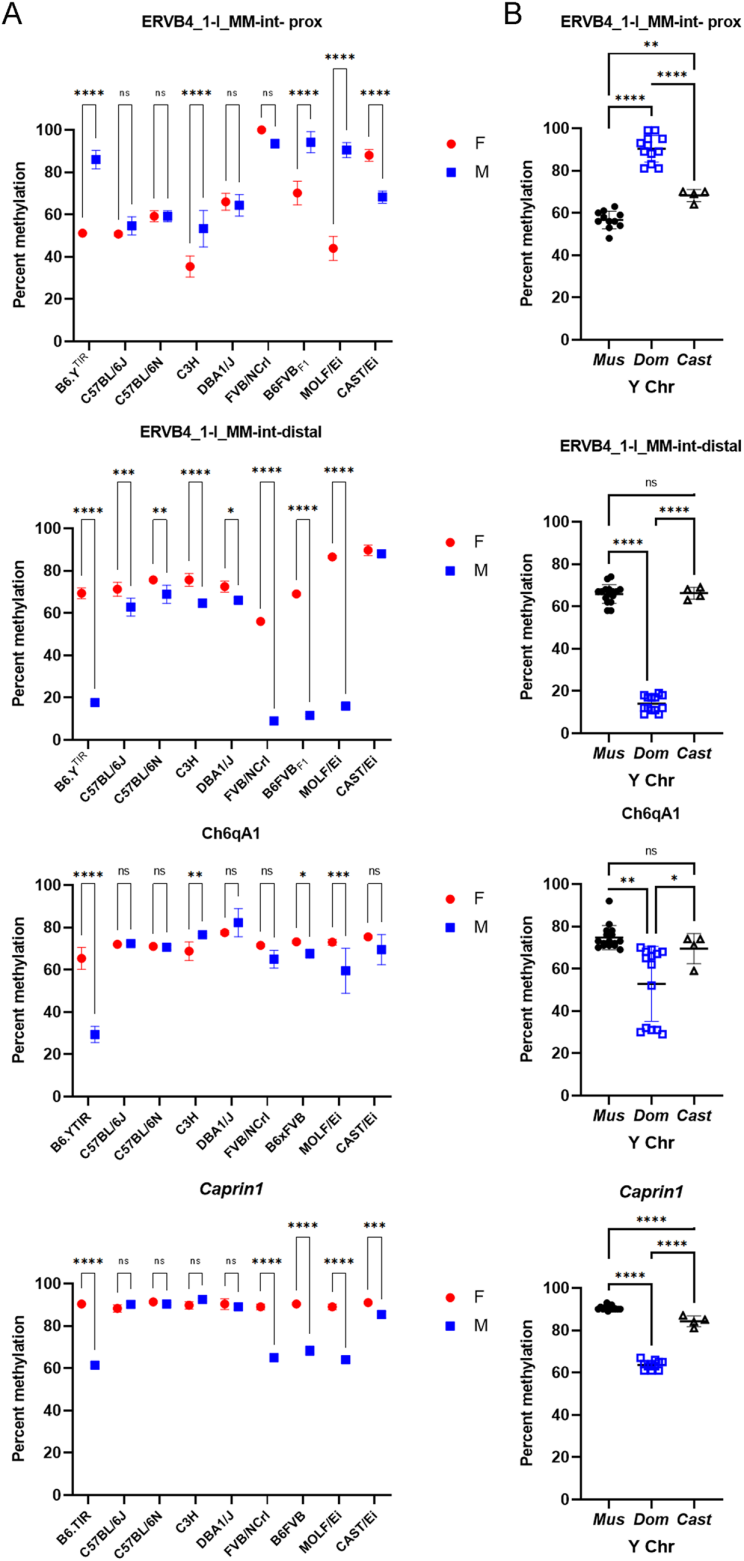
Genetic background and origin of the Y chromosome influence yDMR methylation levels. **A** YDMR methylation levels vary in females and males from different mouse strains. Statistical significance of sex and strain differences was evaluated using two-way ANOVA followed by multiple testing. **B** YDMR methylation levels differ between mice that carry the *Mus musculus domesticus*, *Mus musculus musculus* or *Mus castaneus* Y chromosomes. For the Ch6qA1 yDMR, B6.Y^TIR^ is different from other strains. Statistical significance of the impact of the origin of the Y chromosome was evaluated using one-way ANOVA followed by multiple testing. For the Ch6qA1 yDMR Brown–Forsythe ANOVA test was used. Error bars show standard deviation. Statistically significant differences between groups shown as asterisks *p-value < 0.05, **p-value < 0.01, ***p-value < 0.005; ****p-value < 0.0001; *ns* non-significant

Sex differences in methylation of the ERVB4-proximal yDMR were found in C3H, B6FVBF1, and MOLF/EiJ mice, but not C57BL/6J, C57BL/6N, and DBA/1J mice. Sex differences in methylation of the ERVB4-distal yDMR were found in most strains (Fig. 2). Variation in methylation among XX samples from different strains reflected the effects of the genetic background in the absence of the Y chromosome, whereas variation among XY samples reflected the influence of genetic background including the Y chromosome (Fig. 2B). The ERVB4 yDMRs showed strain-specific variation in methylation in females. There were also significant differences between males carrying the Y of *musculus* vs *domesticus* origin (Fig. 2B; Table S1). The caveat here is that the ERVB4 yDMRs map to the ERVB4_1_I_MM-int copy of the betaretrovirus 4 transposon in the intron 1 of the agouti gene in the C57BL/6J reference genome (Fig. 1A). Insertion of this retrotransposon is responsible for the black (non-agouti) coat color in C57BL/6J mice and is present in several other strains including C57BL/6N and DBA/1J, but absent in the C3H, FVB/NJ, MOLF/EiJ, and CAST/EiJ mice (Tanave et al. 2019) (Table 2). Bisulfite conversion of DNA that is required for WGBS and pyrosequencing methylation assays reduces the complexity of the DNA sequence, and it is likely that our primers amplify more than one copy of the ERVB4. If our primers amplified only the chromosome 2 target region, we would have observed PCR amplification in B6.Y^TIR^, C57BL/6J, C57BL/6N, DBA/1J, and B6FVBF1 mice but not in C3H, CAST/EiJ, FVB/NCrl, or MOLF/EiJ mice. On one hand, the ERVB4 targets were amplified in all samples. On the other hand, variation in methylation levels that may reflect presence or absence of the ERVB4 copy in the agouti locus was observed in females from different strains. These results demonstrate that our yDMR assays interrogate several copies of the transposon, including the target sequence from the agouti locus in those strains that carry it.

Methylation of the Ch6qA1 yDMR showed sex bias in FVB/NCrl, B6FVBF1, MOLF/EiJ, and C3H mice (Fig. 2A). FVB/NCrl, B6FVBF1 and MOLF/EiJ males had lower methylation levels than females. The bias was reversed in the C3H mice who had higher methylation in males than females, albeit the differences did not exceed 10%. The B6.Y^TIR^ male mice had the lowest methylation levels at the Ch6qA1 yDMR compared to males from other strains (Fig. 2A–B). The *Caprin1* yDMR showed sex bias in methylation in FVB/NCrl, B6FVBF1, MOLF/EiJ, and CAST/EiJ mice.

### Y-linked gene candidates

**Fig. 3:**
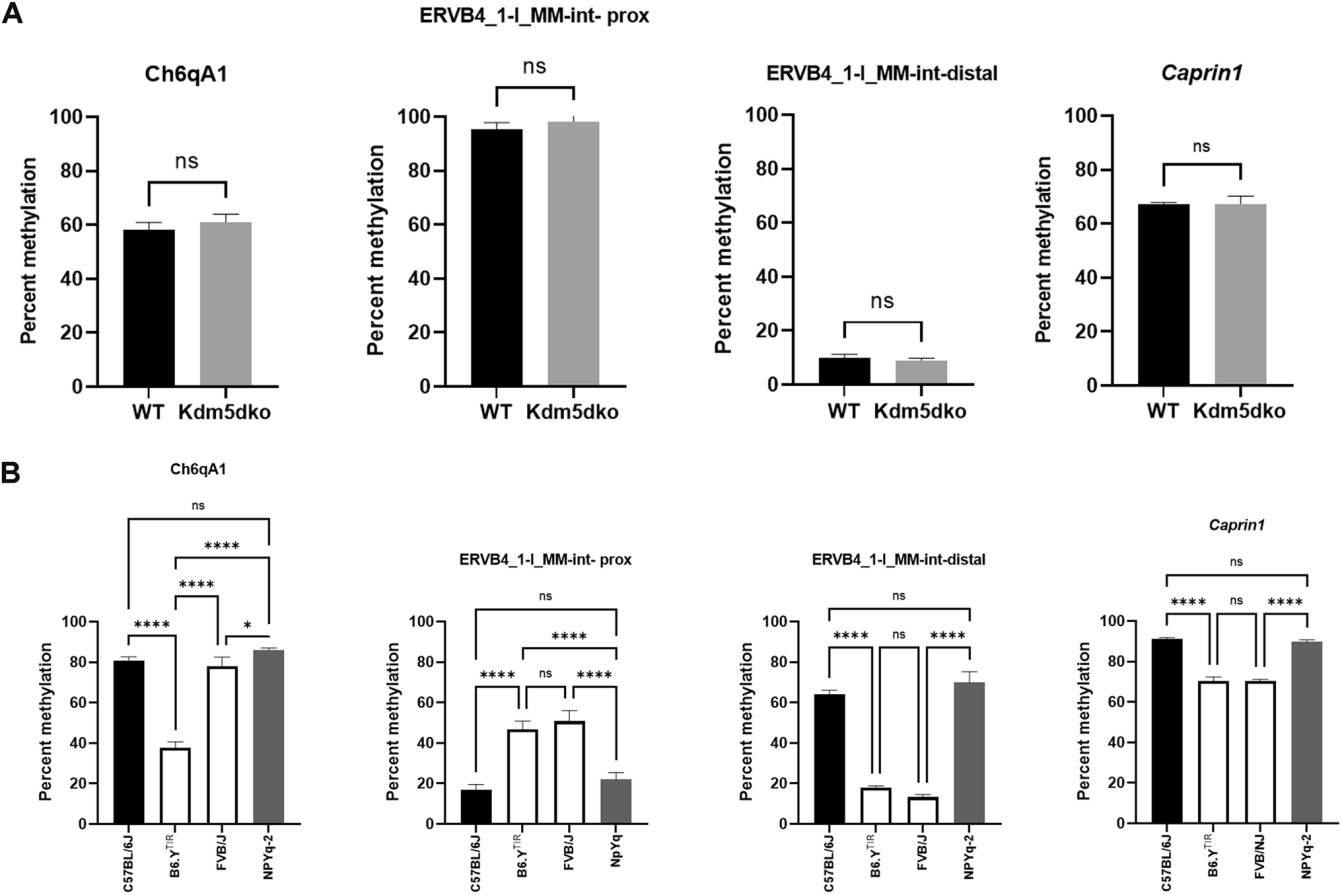
*Kdm5d* and NPYq-2 mutations have no effect on yDMR methylation. **A** Methylation levels of the reporter yDMRs in the livers of wild type (WT) (black bars) and *Kdm5d* mutant (gray bars) mice. **B** Methylation levels of the reporter yDMRs in the testes of C57BL/6J, B6.Y^TIR^, F_1_ (B6 x FVB/NJ) and B6.NPYq-2 mice. Error bars show standard deviation. Statistically significant differences between groups shown as asterisks *p-value < 0.05, **p-value < 0.01, ***p-value < 0.005; ****p-value < 0.0001, *ns* non-significant. Data were analyzed using the non-paired *t*-test (**A**) or one-way ANOVA followed by multiple comparisons (**B**)

We hypothesized that a Y-linked epigenetic modifier or transcription factor(s) was responsible for the Y effect on TE methylation and that genetic variation affecting its function would lead to loss/gain of methylation at yDMRs in XY mice. YDMRs are present in mouse organs derived from the three different germ layers. This may reflect the ubiquitous expression of the Y-linked gene responsible for yDMR methylation or, alternatively, point to a gene that is expressed early in embryonic development driving an early onset of Y-dependent methylation patterns before the germ layers are differentiated. Four Y-linked protein-coding genes residing on the short arm of the mouse Y chromosome, *Kdm5d*, *Uty*, *Ddx3y*, and *Eif2s3y* are expressed in different organs of our mice (Zhuang et al. 2020) and Bauermeister et al. (paper in preparation). If yDMRs are established independently in different somatic cell types, one of these commonly expressed genes must be involved. *Uty* and *Kdm5d*, but not *Eif2s3y* or *Ddx3y* harbor non-synonymous coding polymorphisms between the FVB/NJ and C57BL/6J strains (Mouse Genomes Project, https://www.sanger.ac.uk/data/mouse-genomes-project). The UTY amino acid variation D615N maps outside of the protein domains with known functions (https://beta.uniprot.org/uniprotkb/P79457), whereas the KDM5D variation G213A maps to the disordered domain of the protein (https://beta.uniprot.org/uniprotkb/Q62240). Due to its demonstrated effect on methylation, KDM5D was the most attractive gene candidate. We tested yDMR methylation in the livers and spleens of *Kdm5d* mutant and control mice but found no differences in methylation levels (Fig. 3A). It is worth noting that the knock-out has been generated in the ICR strain of mice, which carry the Y chromosome of *domesticus* origin (Table S2). We next compared the RNA levels of *Kdm5d* in the livers of adult C57BL/6J and B6.Y^TIR^ mice using RT-qPCR. No differences between the two strains were found (Fig. S1A). Hence, we conclude that KDM5D is unlikely to be responsible for the Y-dependent methylation levels.

Next, we asked if the expression levels of the other ubiquitously expressed gene candidates were different between mice with *M. musculus musculus* and *M.musculus domesticus* Y chromosomes. Expression levels of *Eif2s3y*, *Uty*, and *Ddx3y* were retrieved from our RNA-seq data for adult C57BL/6N (AlOgayil et al. 2021) and B6.Y^TIR^ livers (Zhuang et al. 2020) and normalized to *Kdm5d* RNA levels. *Ddx3y* and *Eif2s3y* expression levels were significantly lower, whereas *Uty* levels were significantly higher in B6.Y^TIR^ compared to C57BL/6N livers (Fig. S1B).

The q-arm of the mouse Y chromosome largely consists of amplified repeat units including protein-coding genes that are expressed in the testis and multiple long non-coding RNAs (Comptour et al. 2014; Hughes and Page 2015; Soh et al. 2014). The ampliconic structure of the Yq makes it prone to intrachromosomal rearrangements that poses a challenge for Yq mapping in different strains (Morgan and Pardo-Manuel de Villena 2017). To determine whether the Yq arm harbored gene(s) that influenced methylation, we assayed yDMR methylation in the testes of consomic B6.NPYq-2 mice lacking the Yq arm and several copies of Yp-arm encoded *Rbmy* gene. The remaining Yp-arm was genotyped and its *M. musculus musculus* origin was confirmed (Table S1). No difference in methylation levels between B6.NPYq-2 mice and wild type C57BL/6J mice were found (Fig. 3).

## Discussion

To explore the contribution of the mouse Y chromosome to sexual dimorphism in DNA methylation in somatic cells, we conducted a WGBS data analysis followed by targeted analysis of methylation at several autosomal loci that showed different methylation levels in XY vs XO or XX mice. We show that DNA methylation levels at several loci are associated with the presence of the Y chromosome and independent of the gonadal sex. Firstly, sex-reversed females with two ovaries, hermaphrodites, and males with two testes have the same methylation levels at yDMRs, and secondly, phenotypic males that carry an Y chromosome of *M. musculus musculus* origin have yDMR methylation levels that are not different from those of phenotypic females.

The majority of yDMRs overlap with TEs and most of the TE-related yDMRs have lower methylation in XY compared to XX mice (Table 1). Previous studies have established that gonadal sex has a major impact on DNA methylation in mouse liver (AlOgayil et al. 2021; Hao and Waxman 2021; McCormick et al. 2017; Reizel et al. 2015; Zhuang et al. 2020), whereas the X chromosome dosage predominantly affects DNA methylation of X-linked loci (Zhuang et al. 2020). Here, we report that the Y chromosome influences DNA methylation of certain TEs. We demonstrate that genetic variation in both, the target loci and the Y chromosome itself, must be taken into account when interpreting the results of methylation analyses.

The yDMRs are not liver-specific and are present in different organs. This implies that in contrast to the tissue-specificity of gonadal-sex-dependent methylation (McCormick et al. 2017), the effect of the Y chromosome is not tissue-specific. Silencing of foreign DNA sequences, such as endogenous viruses or transgenes, is a highly conserved fundamental function of DNA methylation that is pivotal for genome integrity [(Conklin et al. 1982; Drahovsky et al. 1979), reviewed in (Bourque et al. 2018; Déléris et al. 2021; Naumova 2013)]. Demethylation and reactivation of endogenous retroviruses is necessary for zygotic genome activation in the early embryo but becomes detrimental in somatic or male germ cells as it facilitates retroviral transposition and hence mutagenesis (Beraldi et al. 2006; Bourc’his and Bestor 2004, reviewed in Jansz 2019). Interestingly, data from human studies support the link between the Y chromosome and TE methylation as certain human cancers, including hepatocellular carcinoma and lung cancer, are characterized by both loss of the Y chromosome and demethylation of TEs in tumor cells (Babaian and Mager 2016; Center et al. 1993; Iskow et al. 2010; Park et al. 2006; Qin et al. 2019; Shitani et al. 2012; Zheng et al. 2019). Examining the associations between human Y-haplotypes, TE methylation, and risks of developing certain diseases with male preponderance, will determine if in humans certain genetic variants of the Y chromosome contribute to both TE demethylation and elevated risk of disease.

Based on our data, we speculate that the mouse Y chromosome harbors a gene(s) that modifies TE methylation levels. Since most yDMRs overlapping with TEs have lower methylation in XY mice compared to XX mice, but XO and XX mice have similar methylation levels, it is a reasonable conjecture that the Y-linked gene product protects TEs from methylation. In an attempt to narrow down the list of gene candidates, we tested mice with a mutation in the *Kdm5d* gene, which was the best gene candidate based on its function, as well as mice lacking the whole Yq arm. These mutations did not affect methylation levels of yDMRs, suggesting that *Kdm5d* or the Yq genes did not contribute to Y-dependent variation in methylation. In principle, regulatory variation in the Y chromosome may influence the expression levels of the putative Y-linked modifier of methylation, which in turn may affect methylation at certain loci. Expression levels of three of the four ubiquitously expressed Y-linked genes, *Eif2s3y*, *Uty*, and *Ddx3y* differ between B6.Y^TIR^ and C57BL/6 mice. Therefore, at this point, we cannot exclude any of these three genes as potential contributors to Y-dependent methylation. It is also worth noting that these Y-linked genes share common regulatory elements: a gene-trap mutation in intron 4 of *Uty* perturbs transcription of the two neighboring genes, *Ddx3y* and *Eif2s3y*, in somatic tissues (Deschepper 2020). Therefore, in future studies targeting exonic mutations that affect the functions of each protein separately may be a more informative approach for identifying the Y-linked modifier of TE methylation.

### Study limitations

One of the major limitations of this study is the lack of complete sequence assemblies of the Y chromosomes from different mouse strains. Sequencing assemblies are hindered by the very structure of the Y chromosome and its ampliconic nature. This adds to the challenges of narrowing down the list of Y-linked gene candidates that contribute to Y-dependent methylation and mapping Y-linked copies of TEs. Moreover, we cannot rule completely out the possibility that our analyses capture TE copies that are located on the Y chromosome itself in addition to autosomal TEs.

The advantage of using WGBS methylation analysis as a starting point is that it permits a relatively unbiased analysis of the mouse methylome and is expected to provide an accurate view of the genomic distribution and repertoire of Y-dependent differentially methylated regions. The caveat here is that the reduced complexity of the DNA sequence after bisulfite conversion may be particularly challenging for mapping repetitive elements. Our data show that after filtering low coverage reads and excluding CGs that overlap with common mouse SNPs, only about one third of the genomic CGs remain available for analysis. We cannot rule out the possibility that the number of TEs containing yDMRs is larger than the one found in our study. Conversely, the possibility of false-positive results is also higher. Therefore, alternative methodologies of genome-wide methylation analysis that do not require bisulfite conversion will provide a more accurate understanding of the impact of the Y chromosome on TE methylation in general and permit a more detailed characterization of its targets.

## Supplementary Information

Below is the link to the electronic supplementary material.

Supplementary file (PDF 267 kb)
