# Supplementary material for: Genetic variation in the Y chromosome and sex-biased DNA methylation in somatic cells in the mouse
Source: Mamm Genome. 2022 Dec 1;34(1):44–55. doi: 10.1007/s00335-022-09970-z (PMC9947081; doi:10.1007/s00335-022-09970-z)
Supplement: Supplementary file 1 — Supplementary file (PDF 267 kb) [file 335_2022_9970_MOESM1_ESM.pdf]

Supplementary material

for

Genetic variation in the Y chromosome and sex-biased DNA methylation in somatic cells in the mouse

Enkhjin Batdorj[1], Najla AlOgayil[1], Qinwei Kim-wee Zhuang[1,2], Jose Hector Galvez[2], Klara Bauermeister[1], Kei Nagata[3], Tohru Kimura[3], Monika A. Ward[4], Teruko Taketo [5,6,7], Guillaume Bourque[1,2], and Anna K. Naumova [1,5,7]

Table S1. Mouse strains and their genotypes in four regions of the Y chromosome

Strain	chrY:724989 C/T<br>*Zfy1*	chrY:1434349 T/C<br>*Usp9y*	chrY:2108567 T/C<br>*Zfy2*	chrY:2663471 A/G<br>*Sry*
B6.NPYq-2	**C**	**C**	**T**	**A**
B6.Y^{TIR}	**C**	**T**	**C**	**G**
C3H/HeH	**C**	**T**	**T**	**A**
C57BL/6J	**C**	**C**	**T**	**A**
C57BL/6N	**C**	**C**	**T**	**A**
CAST/EiJ	**C**	**T**	**T**	**G**
DBA1/J	*C*	*T*	*T*	*A*
FVB/NJ<br>FVB/NCrl	**T**	*T*	**C**	**G**
ICR	**T**	**T**	**C**	**G**
MOLF/EiJ	**T**	*T*	**C**	**G**

DNA from male mice was amplified and PCR products were sequenced to detect single nucleotide polymorphisms in selected Y chromosomal genes. **Bold** denotes our genotyping data; *italics* denotes data from the MGP database.

Table S2. Genotyping primers and primers for RT-qPCR expression assays

Gene/region	Forward primer (5'-3')	Reverse primer (5'-3')	Assay
Sry	GCAGGCTGTAAAATGCCACT	ATGCAGGTGGAAAAGCCTTA	PCR genotyping
chrY:724989 *Zfy1*	TCTGGGTATTTTGAGGCTACTGT	GGCATCCTTGTTCACATCCA	Genotyping by Sanger sequencing
chrY:1434349 *Usp9y*	GCTCTCCTCAGACCTTCCAG	TGGAGTTGCTTGTTTGGTTG	
chrY:2108567 *Zfy2*	TGTTTTGGTACTCTGTCAAGGC	TGCTTTCTTTTGTATTGGCATCT	
chrY:2663471 *Sry*	GCTCTACTCCAGTCTTGCCT	CTGGGATGCAGGTGGAAAAG	
Kdm5d	AGAATCCCAATCTAGAGCGCA	CAGAACCACCTTTTGCCTCC	Expression
Rpl19	GATCATCCGCAAGCCTGTGA	GCATCCGAGCATTGGCAGTA	

Table S3. **List of primers used in pyrosequencing methylation assays**

Assay	*Genic Location*	*Target region*	*Forward primer (5'-3')*	*Reverse primer (5'-3')*	*Sequencing primer (5'-3')*
ERVB4-proximal	Intron 1, enhancer	Chr2:155020006-155020415	TGTGGTATAGTTGTTAAGTAGGAGAGGAT	TACTACTACCCAACAAACCCATAAT	GAGTGATGGAAAATTTAAGT
ERVB4-distal	Intron 1	Chr2:155023201-155023500	AGGTTGTAGAGAGGATTTTAGGTTATG	ACTTAAAACAATTTTTCTAAAAACCTACT	AATTGGTTTTTGAGAATGTTA
Ch6qA1	Intergenic	Chr6:13714801-13715100	GTTATGGAGGTTGGTAATAATGTT	ACCAATAAAAATCAAACAATTCACAAA	GGGGAATATATAGAAAGATATAAAA
Caprin1	Exon 9	Chr2:103766401-103766700	TTAAGTAAGGGTGAGGAATTT	AAAAAAACTATTTTCATCCCATAAAAACAA	TGTATTGTGTGTAAGGTTT
Ch16	Intergenic	Chr16:21107101-21107400	GTTTGTTAAGATTGATAATGGATTAGTTTA	CTCAACAATACCCTATCCTTAAAA	TTTAAAAATTTTAGTAGTTTTGT

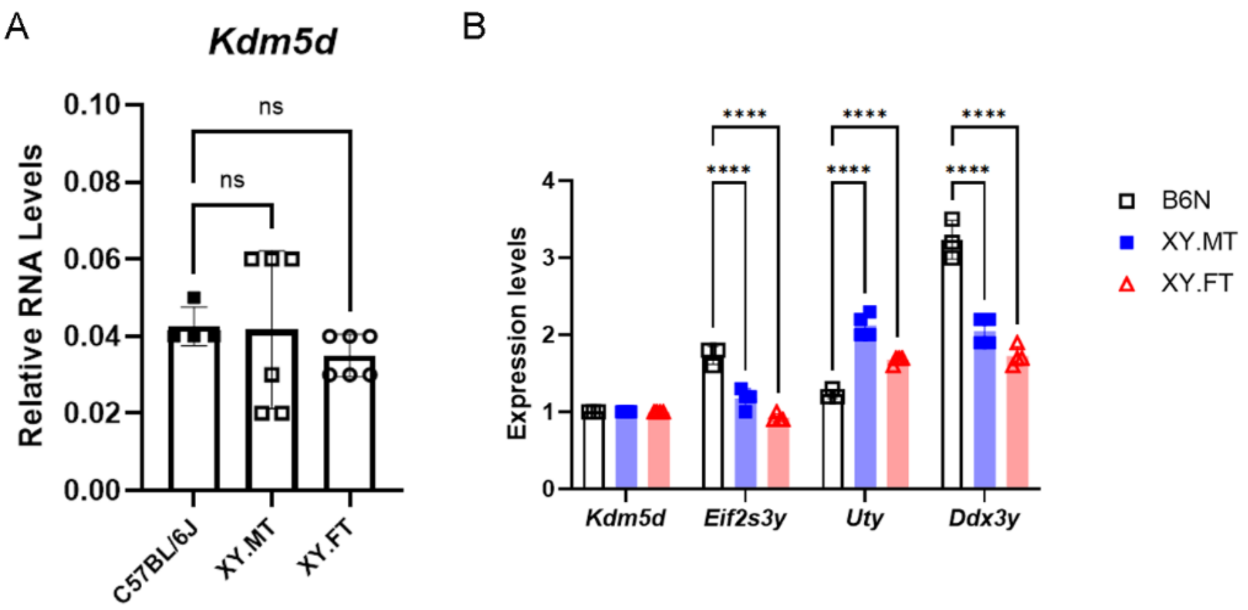

Figure S1. Comparison of expression levels of Y-linked genes in mice with *M. musculus musculus* and *M. musculus domesticus* Y chromosomes. A. RNA levels in livers of C57BL/6J male mice (n=4) and B6.Y^{TIR} males (XY.MT, n=6) and females (XY.FT, n=6). Data from RT-qPCR normalized by *Rpl19* levels. B. RNA levels in livers of wild type C57BL/6N males (n=3) and B6.Y^{TIR} males (XY.MT, n=4) and females (XY.FT, n=4). Data extracted from previously published RNA-seq experiments conducted in our labs and normalized by *Kdm5d* levels in each group, separately.
